# Development of real-time PCR assays for evaluation of immune response and parasite load in golden hamster (*Mesocricetus auratus*) infected by *Leishmania* (Viannia) *braziliensis*

**DOI:** 10.1186/s13071-016-1647-6

**Published:** 2016-06-27

**Authors:** Raquel Peralva Ribeiro-Romão, Andrea Franco Saavedra, Alda Maria Da-Cruz, Eduardo Fonseca Pinto, Otacilio C. Moreira

**Affiliations:** Laboratório Interdisciplinar de Pesquisas Médicas, Instituto Oswaldo Cruz (FIOCRUZ/RJ), Rio de Janeiro, Brazil; Laboratório de Biologia Molecular e Doenças Endêmicas, Instituto Oswaldo Cruz (FIOCRUZ/RJ), Rio de Janeiro, Brazil

**Keywords:** Golden hamster, *Mesocricetus auratus*, *Leishmania* (*Viannia*) *braziliensis*, Cytokine gene expression, Parasite load, Real time PCR, RT-qPCR, qPCR

## Abstract

**Background:**

Cutaneous leishmaniasis (CL) is a neglected disease with a broad spectrum of clinical manifestations, ranging from small cutaneous nodules to severe mucosal tissue destruction. *Leishmania* (*Viannia*) *braziliensis* is the main species attributed to CL in the Americas. However, studies of experimental infection are limited in the murine model due to the self-resolutive pattern of the disease. Previously, our group demonstrated that the hamster model reproduces many of the clinical and histopathological features observed in humans. Herein, we standardized a RT-qPCR gene expression assay to evaluate a panel of immunological markers and a qPCR assay in order to quantify with high sensitivity and reproducibility the parasite load in skin lesions.

**Methods:**

Hamsters were intradermally infected in the footpad with 10^5^ promastigotes of *L.* (*V*.) *braziliensis* and 110 days post-infection skin lesions and popliteal lymph nodes were removed for RNA and DNA extraction, both from the same tissue fragment. Gene expression of IFN-ɣ, IL-10, TGF-β TNF, IL-4, IL-6, iNOS and arginase were measured using non-infected animal tissue as a calibrator. Parasite load was quantified from DNA extracted from lesions by qPCR targeting *Leishmania* kDNA and normalized by hamster GAPDH, using a SYBR Green-based absolute quantification methodology.

**Results:**

A relative quantification RT-qPCR assay was standardized for the evaluation of mRNA levels from skin and lymph node samples of golden hamsters, with PCR efficiencies ranging from 92.3 to 116.4 %. In uninfected animals, higher basal mRNA levels in lymph nodes were observed for IFN-ɣ, TGF-β, TNF and IL-4 (111.4 ± 92.2; 5.6 ± 1.2; 5.3 ± 1.7; and 60.3 ± 26.8, respectively) in comparison to skin. In golden hamsters infected with *L.* (*V*.) *braziliensis*, an increase in the expression of all immunological markers evaluated was observed, ranging from 2.7 ± 0.2 for TGF-β to 1018.5 ± 809.0 for iNOS in skin lesions, and 2.4 ± 1.6 for TGF-β to 600.2 ± 666.4 for iNOS in popliteal lymph nodes. Interestingly, significantly higher levels of IFN-ɣ, TNF and IL-10 mRNA were observed in skin in comparison to lymph nodes, while a lower significant level of arginase mRNA was observed in skin. In parallel, parasite loads were quantified by qPCR from the skin lesions of infected animals, ranging from 27.0 to 6647.0, with a median of 553.4 (416.7–1504.0) parasites/mg skin equivalents, whereas lesion size varied from 0.3 to 3.1 mm. Despite the tendency of larger lesions to present higher parasite load, the correlation observed was not statistically significant.

**Conclusions:**

In this study, we describe for the first time a sensitive, reproducible and cheaper molecular assay to quantify from the same tissue fragment the gene expression of immunological markers and the parasite load in skin lesions, observing a mixed profile of immune response in the hamster model infected by *L.* (*V*.) *braziliensis*.

**Electronic supplementary material:**

The online version of this article (doi:10.1186/s13071-016-1647-6) contains supplementary material, which is available to authorized users.

## Background

Leishmaniasis is a protozoan vector-borne disease caused by *Leishmania* spp., highlighted as the first neglected tropical disease ranked by disability-adjusted life years (DALYs) [1]. Cutaneous leishmaniasis (CL) is present in 98 countries on five continents with an annual incidence of over 220,000 notified cases, but with an estimated annual incidence ranging from 0.7 to 1.2 million cases [2]. In the Americas, *L.* (*Viannia*) *braziliensis* is one of the species of major importance in public health due to its prevalence, therapeutic failure and the possibility of developing the mucosal form of the disease, which is more severe and difficult to treat [3–5]. Despite numerous studies conducted in recent decades, there is still no vaccine licensed for use in humans and the treatment remains mostly based on pentavalent antimonials [4]. These data reinforce the need to investigate new drugs and vaccine candidates for the control and prophylaxis of CL.

The study of *L. braziliensis* infection is limited by the difficulty of an appropriate experimental model, since most mouse strains are resistant to infection by this species [6, 7]. The golden hamster (*Mesocricetus auratus*) is the most susceptible model for the infection by species of the subgenus *Viannia*, reproducing many of the clinical and histopathological features observed in the human disease [8–10]. Furthermore, its outbred genetic background, which highlights individual characteristics, reproduces the heterogeneity of clinical outcomes observed in human CL [11]. Nevertheless, few studies on drugs [12–15] and vaccines [16, 17] against CL are performed in the hamster model.

Research in this model is hampered by the lack of available reagents such as antibodies to cell markers and cytokines, since evaluation of factors related to immune response are of great importance to understand the immunopathogenesis of the disease [18–20]. Most of what we know about the immune response to *Leishmania* in hamsters is due to infection by *L. donovani* and *L. panamensis* through molecular approaches [9, 18, 21, 22]. In this sense, the development of immunological and molecular tools to analyse *L. braziliensis* infection in the hamster model is of great importance to understand the host-parasite relationship, in an attempt to explain the phenomena that occur in human disease, and to establish parameters to evaluate vaccine and new drugs candidates.

Besides the evaluation of the immune response, the development of a technique that allows a precise quantitation of parasites in different tissues is extremely important to evaluate the impact of parasite load in experimentally infected hamsters. Real time PCR has high sensitivity, accuracy and reproducibility to quantify tissue parasitism being an essential tool for assessing parasite load in the hamster model after experimental treatment or immunization with potential vaccine candidates [23]. Recently, it was demonstrated that the real time PCR targeting the *Leishmania* kDNA minicircles presents higher sensitivity compared to traditional methods or other targets [24].

In this study, we standardized and validated a highly sensitive and less expensive SYBR Green-based RT-qPCR assay to assess a panel of immunological markers related to human immunopathogenesis of cutaneous leishmaniasis and a qPCR assay to quantitate parasite load from hamsters infected with *L. braziliensis*. To our knowledge, this is the first report that shows a comprehensive assessment of the immune response in the hamster model of *L. braziliensis* infection as well as the quantitation of parasite load by qPCR simultaneously from the same tissue fragment.

## Methods

### Animals and ethics statements

Twenty-one outbred adult female (6–8 week-old) Syrian golden hamsters (*M. auratus*), weighing 80 to 90 g, were obtained from the animal facilities of the Fundação Oswaldo Cruz (FIOCRUZ). This study was approved by the Ethics Committee on Animal Use (CEUA) of FIOCRUZ, with protocol number LW 11/11.

### Parasites and infection

*L.* (*V*.) *braziliensis* (MCAN/BR/98/R619) was maintained in Schneider’s Drosophila medium (Sigma Chemical Co., USA) supplemented with 20 % fetal bovine serum (Life Technologies, Brazil), L-glutamine (1 mM; Life Technologies, Brazil), and antibiotics (200 g/ml penicillin and 20 g/ml streptomycin; Sigma Chemical Co., USA) at 26 °C. Parasites in the stationary growth phase from the third in vitro passage were washed in sterile phosphate-buffered saline (PBS) and counted on a Neubauer chamber. The inoculum was prepared containing 1 × 10^5^ parasites in a total volume of 20 μl of PBS for intradermal inoculation into the dorsal hind paw of hamsters. Lesion sizes were measured 110 days post-infection with a dial caliper (Mitutoyo, America Corporation, São Paulo, Brazil) and expressed as the difference between the thickness of the infected and uninfected paws.

### RNA and DNA extraction

Immunological markers gene expression and parasite load were quantitated from the same skin lesion fragment using a combination of TRIzol® (Invitrogen, California, USA) and RNeasy® mini kit (Qiagen, Austin, Texas, USA) for RNA extraction, and TRIzol® for DNA extraction. For that, 20 to 30 mg of skin from the inoculation site or draining popliteal lymph node of infected and uninfected animals were immersed in TRIzol® and lysed with the homogenizer Ultra-Turrax tissue Dispenser (IKA, Wilmington, USA) for 30 s. According to the TRIzol® manual, addition of chloroform followed by centrifugation separates the solution into an aqueous phase and an organic phase. RNA remains exclusively in the aqueous phase. After separation of the aqueous phase, the RNA is recovered by precipitation with isopropyl alcohol. After removal of the aqueous phase, sequential precipitation with ethanol yields DNA from the organic phase.

Samples homogenized in 1 ml TRIzol were treated for 30 min at 56 °C with 10 μl of Proteinase K (10 mg/ml) (Invitrogen, California, USA). Following this, 200 μl of chloroform were added to the lysate, and the organic and aqueous phases were separated by centrifugation at 12,000 × *g* for 15 min, at 4 °C. From the aqueous phase, RNA was extracted using the RNeasy mini Kit and resuspended in 30 μl of elution buffer, for the immunological markers gene expression analysis, following the manufacturer’s protocol. From the remaining organic phase, DNA was extracted for the quantitation of parasite load, also following manufacture’s protocol. Briefly, DNA was precipitated in 0.3 ml of 100 % ethanol. After precipitation by centrifugation, DNA was washed twice in 1 ml sodium citrate solution (0.1 M) in 10 % ethanol (with 30 min interval between each wash). Then, DNA was washed in 1.5 ml of 75 % ethanol, the pellet was air-dried and dissolved in 400 μl sodium hydroxide solution (8 mM). DNA and RNA were stored at -20 °C and -80 °C until use, respectively.

### Reverse transcription

RNA samples were treated with RQ1 RNase-free DNase (Promega Corporation, Madison WI, USA), quantitated using the Spectophotometer Pico 200 (Picodrop Ltd., Saffon Walden, United Kingdom) and kept at -80 °C until use. RNA (2 μg) was reverse transcribed using a High Capacity Reverse Transcription kit (Applied Biosystems, Foster City, CA, USA). The cDNA concentration was quantitated using Qubit® ssDNA Assay Kit (Life Technologies, Eugene, Oregon, USA) and adjusted to a final concentration of 10 ng/μl.

### IFN-γ, TNF, IL-6, iNOS, IL-10, TGF-β, IL-4 and arginase gene expression analysis by RT-qPCR

The evaluation of the immunological markers mRNA levels in golden hamsters infected with *L.* (*V*.) *braziliensis* were based on sequences of primers already described [11, 18, 25, 26] or designed for this study, according to Table 1. For real-time PCR assays, 2 μl cDNA (at 10 ng/μl) were used in a final reaction volume of 10 μl, with 5.0 μl of Power SYBR Green PCR Master Mix 2X (Life Technologies, CA, USA), 100 nM of forward and 100 nM of reverse primers (except for the IL-4 primers, where 200 nM of Forward and Reverse primers were used), in a ViiA7 Real-Time PCR System (Applied Biosystems, Foster City, CA, USA), in 384 well plates. PCR cycling conditions were: a first step at 95 °C for 10 min, followed by 40 cycles at 95 °C for 15 s and 60 °C for 1 min. To check for the primers specificity, melting curves were generated after the 40 cycles. Gene expression was calculated by relative quantitation using the comparative Ct method (ΔΔCt), as previously described [27], with threshold set at 0.02. As reference genes, the housekeeping GAPDH and γActin genes were used (Table 1). Gene expression was expressed as fold change (2^-ΔΔCt^), in relation to samples from uninfected hamsters, used as calibrators. For the validation of constitutive expression of reference gene candidates and gene expression analysis, the Expression Suite Software (Life Technologies) was used.

**Table 1:** Primers and standard curve parameters for immunological marker amplification in golden hamsters infected by *Leishmania* (*V*.) *braziliensis*

Golden hamster gene target	Primer sequences	Reference	Amplicon length	Slope	Intercept	Coefficient of linearity (r^2^)	Amplification efficiency (%)
GAPDH	Fw 5′-GGT TGC CAA ACC TTA TCA GAA ATG-3′	Ribeiro-Romão et al. [11]	194 bp	-3.30	20.03	0.98	100.7
	Rv 5′-TTC ACC TGT TCC ACA GCC TTG-3′						
ɣ Actin	Fw 5′-ACA GAG AGA AGA TGA CGC AGA TAA TG-3′	Espitia et al. [18]	70 bp	-3,28	20.06	0.99	101.7
	Rv 5′-GCC TGA ATG GCC ACG TAC A-3′						
IFN-ɣ	Fw 5′-TGT TGC TCT GCC TCA CTC AGG-3′	Espitia et al. [18]	130 bp	-3.48	26.03	0.99	93.4
	Rv 5′-AAG ACG AGG TCC CCT CCA TTC-3′						
TGF-β	Fw 5′-GGC TAC CAC GCC AAC TTC TG-3′	Espitia et al. [18]	81 bp	-3.52	27.11	0.99	92.3
	Rv 5′-GAG GGC AAG GAC CTT ACT GTA CTG-3′						
TNF	Fw 5′-TGA GCC ATC GTG CCA ATG-3′	Espitia et al. [18]	79 bp	-3.33	24.81	0.99	99.5
	Rv 5′-AGC CCG TCT GCT GGT ATC AC-3′						
IL-10	Fw 5′-GGT TGC CAA ACC TTA TCA GAA ATG-3′	Espitia et al. [18]	194 bp	-3.21	29.66	0.99	104.7
	Rv 5′-TTC ACC TGT TCC ACA GCC TTG-3′						
IL-4	Fw 5′-CCA CGG AGA AAG ACC TCA TCT G-3′	Zivcec et al. [25]	72 bp	-2.98	31.06	0.98	116.4
	Rv 5′-GGG TCA CCT CAT GTT GGA AAT AAA-3′						
IL-6	Fw 5′-GGA CAA TGA CTA TGT GTT GTT AGA A-3′	This study	99 bp	-3.23	28.85	0.97	103.8
	Rv 5′- AGG CAA ATT TCC CAA TTG TAT CCA G-3′						
iNOS	Fw 5′-TGA GCC ACT GAG TTC TCC TAA GG-3′	Osorio et al. [26]	93 bp	-3.54	29.71	0.95	92.7
	Rv 5′-TCC TAT TTC AAC TCC AAG ATG TTC TG-3′						
Arginase	Fw 5′-ACC TAT GTG TCA TTT GGG TGG A-3′	Osorio et al. [26]	163 bp	-3.38	20.57	0.99	97.5
	Rv 5′-GCA GAT ATG CAG GGA GTC ACC-3′						

### Parasite load by Quantitative Real-Time PCR (qPCR)

The measurement of parasite load by qPCR was performed by absolute quantitation, based on a standard curve produced from DNA samples extracted from fragments of hamster skin, artificially infected with promastigote forms of *L. braziliensis*. For this, 3 mg of skin from the dorsal hind paw of a hamster (uninfected animal) were spiked with 10^5^ promastigotes from *L. braziliensis*, and DNA was extracted as described. Serial dilutions of 1:10 were made with Tris-EDTA buffer (TE) to produce seven points of the standard curves, ranging from 10^5^ to 0.5 parasite equivalents, or from 3 to 3 × 10^-6^ mg skin equivalents. For parasite quantitation, primers targeting the conserved regions of kinetoplastid DNA minicircles (kDNA) were used [28], at 50nM and 50nM (forward and reverse primers, respectively). Prior to the qPCR assay, hamster DNA samples were diluted 1:500 to better suit the parasite standard curve. The reactions were performed in a ViiATM 7 Real-Time PCR System (Applied Biosystems, USA) with 5 μl of the Power SYBR Green master mix 2X, in a final volume of 10 μl per well. PCR cycling conditions were: a first step at 95 °C for 10 min, followed by 40 cycles at 95 °C for 15 s and 60 °C for 1 min for gene expressions assay, and 64 °C for parasite load assay, respectively. To check for the primers specificity, melting curves were generated after the 40 cycles. The parasite load was calculated by the (*L. braziliensis* equivalents/hamster skin mass equivalents) ratio and expressed as “parasite load” (parasites eq./mg skin).

### Statistical analysis

All RT-qPCR and qPCR assays were performed in biological quintuplicate, and experimental duplicates or triplicates. Results were expressed as the means ± standard deviations. Statistical tests from the ΔCt values (student t-test or Mann-Whitney rank sum test, and Analysis of Variance) were performed with GraphPad Prism software version 5.00 for Windows (GraphPad Software, San Diego, CA, USA) or SigmaPlot v12.0 software (Systat Software, Inc).

## Results

### Hamster tissue mRNA expression by RT-qPCR

The gene expression analysis of several cytokines and inflammation-related genes can be a challenge that demands a well-refined standardization of PCR, in order to achieve the best amplification signals, especially in control samples. So, this study began with the standardization of RT-qPCR assay using SYBR Green fluorophore, mutual to all targets. At 60 °C annealing/extension temperature in the PCR cycle, we performed a primer concentration curve, where 100 nM × 100 nM for the forward and reverse primers presented the best performance, taking into account the Ct values, primer-dimer formation and reagents economy. For the IL-4 target, the best primer concentrations achieved were 200 nM × 200 nM (Additional file 1: Table S1). Furthermore, the cDNA concentration of 10 ng/ul (20 ng/reaction) allowed amplification of all targets, including the samples of uninfected hamsters.

In order to achieve and report the experimental conditions and assay characteristics to estimate with accuracy the mRNA levels, the MIQE (Minimum Information for Publication of Quantitative Real-Time PCR Experiments) guideline [29] was followed in this study. Accordingly, robust and precise qPCR assays are usually correlated with high PCR efficiency, which is calculated from the slopes of calibration curves following Efficiency = 10^1^/slope 1. In addition, the dynamic range over which the reaction was linear (the highest to the lowest quantifiable cDNA amount, established by means of a calibration curve) was determined for each target.

**Fig. 1:**
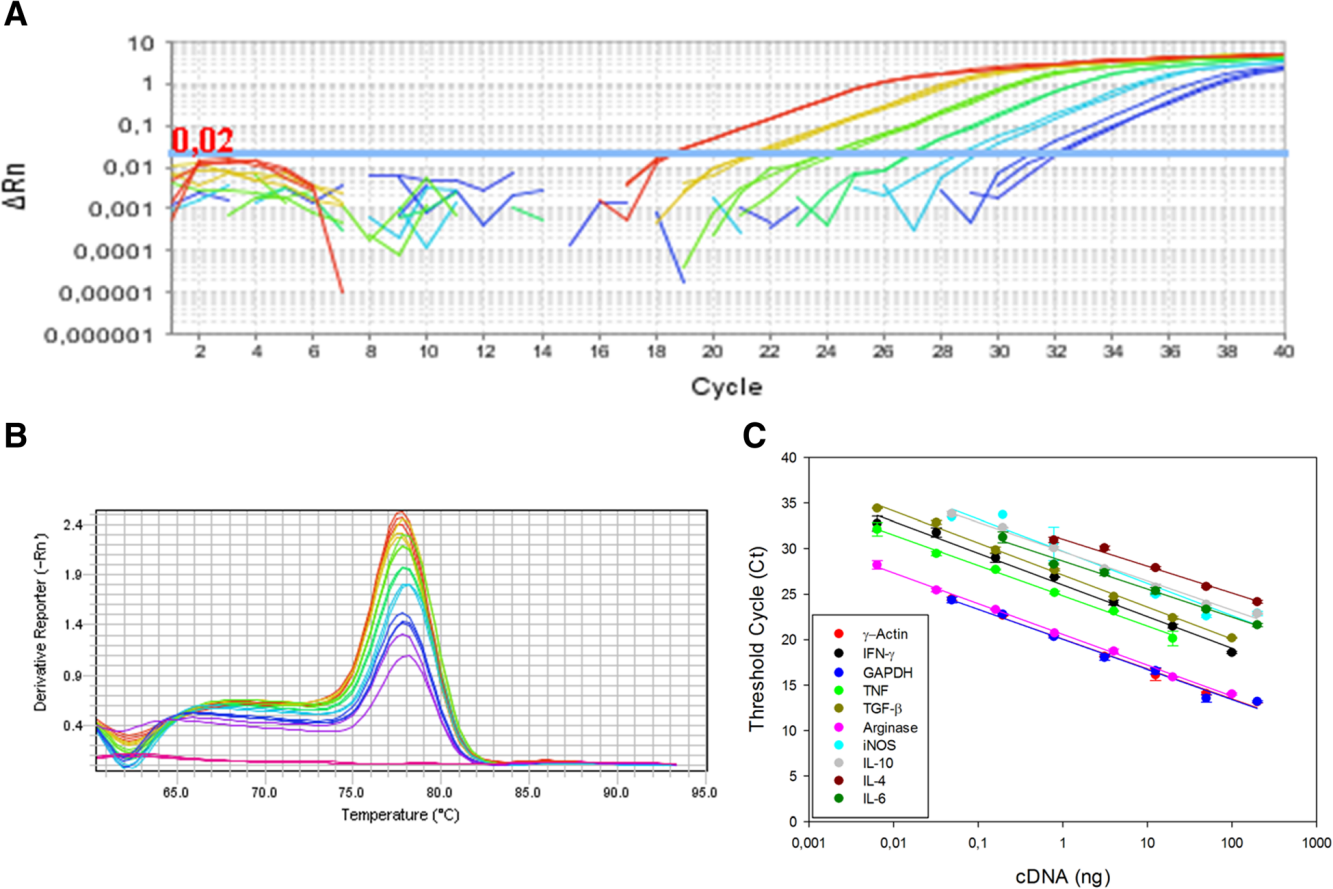
Standardization of RT-qPCR assays for gene expression analysis of immunological markers. Golden hamsters were infected with 10^5^ *Leishmania* (*V*.) *braziliensis* promastigotes, for 110 days. The panel shows a representative amplification plot with the fluorescent signal magnitude (**a**) and melting curve indicating the reaction specificity, observed through a single peak in each curve (**b**). Calibration curves of γ-Actin, IFN-γ, GAPDH, TNF, TGF-β, Arginase, iNOS, IL-10, IL-4 and IL-6 target genes from golden hamster indicates the linearity of the reaction (**c**). RT-qPCR assays were performed using SYBR Green fluorophore, as described in Methods section

Thus, to estimate PCR efficiency and to observe the dynamic extension for gene expression quantitation, 1:5 serial dilution curves (calibration curves) of cDNAs (from 100 ng) were compared (Fig. 1a and c). Moreover, after the PCR with SYBR Green fluorophore, the melt curves for all targets indicated the specificity of the reactions (Fig. 1b), represented by a single peak in each curve. In contrast, no peaks were observed in the Negative Template Control (represented by pink lines), indicating the absence of primer-dimers in the PCR. For most targets, amplification was linear from 100 to 0.0064 or 0.0032 ng of cDNA. For IL-4 and IL-6 targets, which present lower expression levels, 1:4 serial dilution curves from 200 ng cDNA were performed. To these targets, amplification was linear to 0.78 and 0.20 ng of cDNA, respectively. Table 1 shows the PCR efficiency for each target gene, which varied from 92.3 % (TGF-β target) to 116.4 % (IL-4 target). For the reference genes, efficiencies were 100.7 and 101.7 % to GAPDH and ɣ-actin, respectively.

**Fig. 2:**
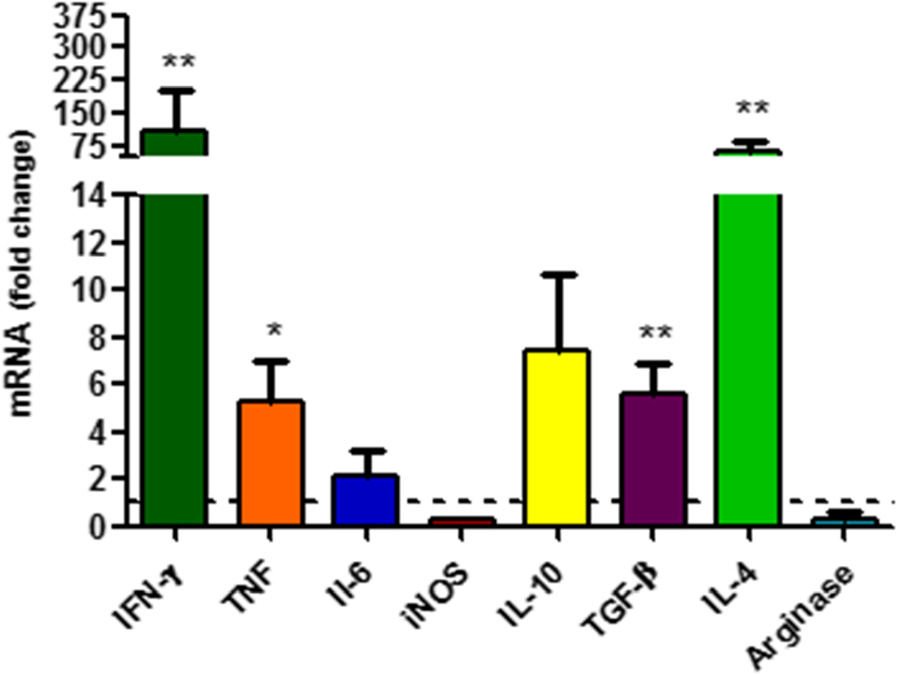
Gene expression of immunological markers in uninfected golden hamsters. The graphic indicates the fold change of mRNA levels in lymph node in comparison to skin. The relative quantification was performed by the comparative Ct method (△△Ct), using skin from uninfected hamster as calibrator (Fold change = 1), as indicated by the dotted line. Horizontal bars represent the mean ± standard deviation of four biological replicates. (*P < 0.05; **P < 0.001; IFN-ɣ: t-test: *t*_(7)_ = 5.94, *P* < 0.0001), IL-4: t-test: *t*_(5)_ = 8.91, *P* < 0.0001, TGF-β: t-test: *t*_(5)_ = 11.26, *P* < 0.0001 and TNF: t-test: *t*_(5)_ = 5.23, *P* = 0.003)

Following the standardization, the basal gene expression of immunological markers between skin and lymph node samples were compared, from uninfected animals. In general, a higher basal gene expression in lymph node was observed in comparison to skin, with statistical significance for IFN-ɣ, with fold change mean of 111.4 ± 92.2 (t-test: *t*_(7)_ = 5.94, *P* < 0.0001), IL-4: 60.3 ± 26.8 (t-test: *t*_(5)_ = 8.91, *P* < 0.0001), TGF-β: 5.6 ± 1.2 (t-test: *t*_(5)_ = 11.26, *P* < 0.0001) and TNF: 5.3 ± 1.7 (t-test: *t*_(5)_ = 5.23, *P* = 0.003) (Fig. 2). The magnitude of amplification and fluorescent signal detection, evaluated through the threshold cycles (Ct), were different between the targets (Additional file 2: Table S2). The IFN-ɣ and IL-10 in the skin, and iNOS in the skin and lymph node, presented a low basal gene expression (Ct higher than 31). In contrast, TNF, IL-6 and IL-4 in skin, and IFN-ɣ, IL-10, IL-4 and IL-6 in lymph node presented an intermediary basal expression (Cts between 23 and 30). High basal expressions were observed in TGF-β and arginase genes in skin and lymph node, and TNF in lymph node, with Cts lower than 21.

**Fig. 3:**
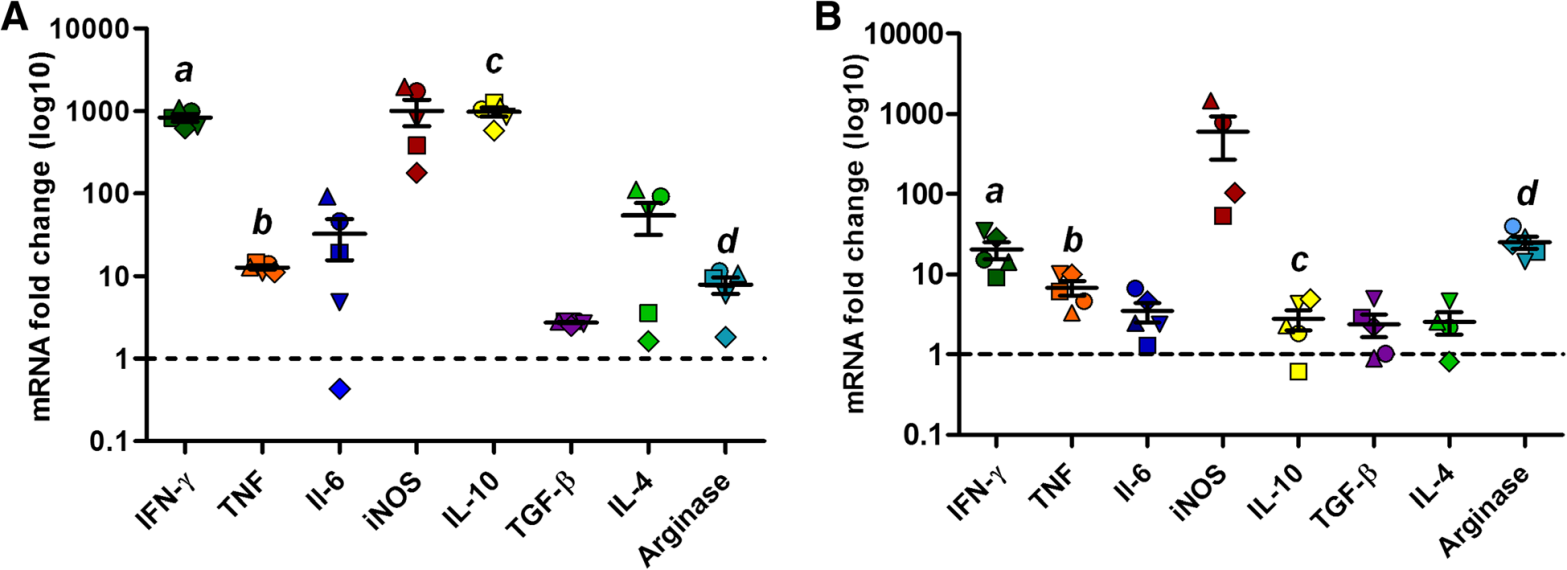
Gene expression of immunological markers in golden hamsters infected with *Leishmania* (*V*.) *braziliensis.* The graphics show the fold change of mRNA levels in skin (**a**) and lymph node (**b**) of golden hamsters infected with 10^5^ *Leishmania* (*V*.) *braziliensis* promastigotes, 110 days post-infection. The relative quantification was performed by the comparative Ct method (△△Ct), using lymph node or skin from uninfected animals, respectively, as calibrator (Fold change = 1), as indicated by the *dotted line*. Each symbol represents a sample from a different animal, and each gene target is represented by a different colour. The median and interquartile intervals are indicated in the graphics. The letters *a*, *b*, *c* and *d* indicates statistical differences between mRNA levels in skin and lymph node (IFN-ɣ: Rank Sum Test: *T*_(5,5)_ = 40, *P* = 0.008, TNF: t-test: *t*_(8)_ = 3.72, *P* = 0.003, IL-10: Rank Sum Test: *T*_(5,5)_ = 40, *P* = 0.008, arginase: t-test: *t*_(8)_ = -3.69, *P* = 0.006)

Then, RT-qPCR assay was applied to assess gene expression in the skin and popliteal lymph node from hamsters infected with 1 × 10^5^ *L. braziliensis* promastigotes 110 days post-infection, in order to investigate the immune response profile. An increase was observed in the gene expression of all cytokines and enzymes evaluated in skin of infected hamsters, in comparison to uninfected animals, with a mean fold change of 840.9 (± 209.2) to IFN-ɣ gene, 12.8 (± 1.6) to TNF, 32.6 (± 37.9) to IL-6, 1018.5 (± 809.0) to iNOS, 983.8 (± 270.9) to IL-10, 2.7 (± 0.2) to TGF-β, 54.1 (± 50.0) to IL-4 and 7.9 (± 4.0) to arginase (Fig. 3a). Popliteal lymph node also presented an increase in the gene expression for all targets, with an average of 20.5 (± 10.8) fold change in IFN-ɣ gene expression, 6.9 (± 3.1) to TNF, 3.5 (± 2.1) to IL-6, 600.2 (± 666.4) to iNOS, 2.8 (± 1.8) to IL-10, 2.4 (± 1.6) to TGF-β, 2.6 (± 1.6) to IL-4 and 25.3 (± 9.7) to arginase (Fig. 3b). A significant difference was observed between skin and popliteal lymph node gene expression of infected hamsters: the IFN-ɣ (Rank Sum Test: *T*_(5,5)_ = 40, *P* = 0.008), TNF (t-test: *t*_(8)_ = 3.72, *P* = 0.003) and IL-10 (Rank Sum Test: *T*_(5,5)_ = 40, *P* = 0.008) genes presented increased expressions in skin compared to lymph node, whereas more arginase was expressed in lymph nodes (t-test: *t*_(8)_ = -3.69, *P* = 0.006).

### *Leishmania* (*Viannia*) *braziliensis* parasite load by quantitative real time PCR (qPCR)

To standardize the parasite load quantitation by qPCR using the SYBR Green fluorophore, annealing temperatures from 60 to 64 °C (Additional file 3: Table S3) and primer concentrations from 10 to 300 nM (Additional file 4: Table S4) were tested. Based on the Ct values and specificity (primer-dimer formation), the annealing/extension temperature of 64 °C, and 50 nM of each primer were defined.

**Fig. 4:**
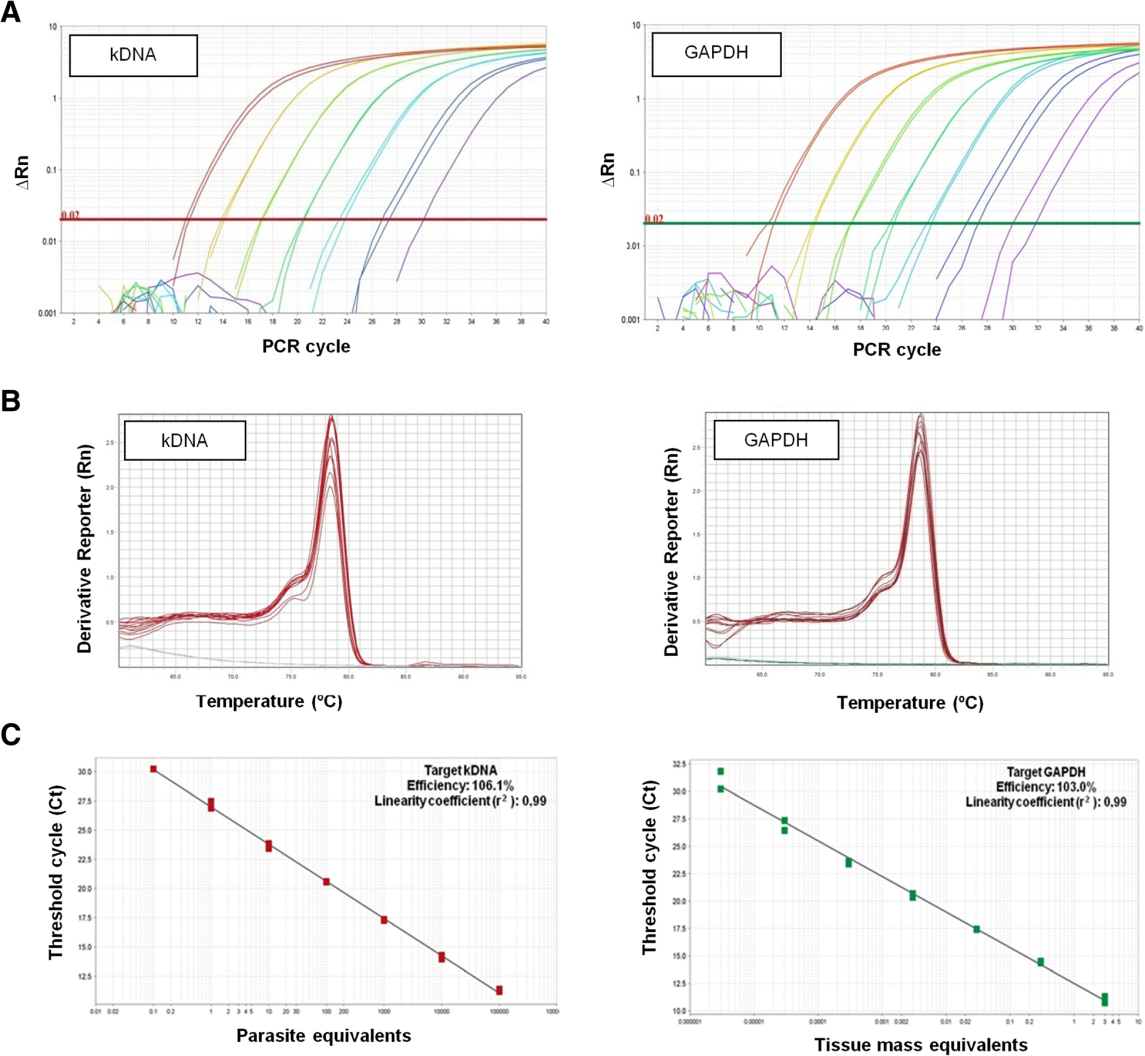
Standardization of qPCR assays for parasite load quantitation from skin lesions of golden hamsters infected with *Leishmania* (*V*.) *braziliensis.* Golden hamsters were infected with 10^5^ *Leishmania* (*V*.) *braziliensis* promastigotes, for 110 days. The panel shows representative amplification plots with the fluorescent signal magnitude for parasite kDNA and golden hamster GAPDH targets (**a**) and *melting curve* indicating the reaction specificity, observed through a single peak in each *curve* (**b**) Standard curves for parasite kDNA and golden hamster GAPDH targets indicate the dynamic extension, PCR efficiency and linearity coefficient of the reaction (**c**) To run the experiments, DNA and RNA were extracted from the same sample and qPCR assays were performed using SYBR Green fluorophore, as described in Methods section

For the absolute quantitation by qPCR, standard curves were constructed ranging from 10^5^ to 0.5 parasite equivalents and 3 to 3 × 10^-6^ mg skin equivalents. According to this assay, it was possible to quantitate up to 0.5 parasite and 3 × 10^-6^ mg hamster skin equivalents, and no lack of specificity or significant primer-dimer formation was observed through melt curve analysis (Fig. 4a and b). An efficiency of 106.1 % could be observed for the *L. braziliensis* kDNA target, with a linearity coefficient of 0.99, and the efficiency of 103.0 % could be observed for golden hamster GAPDH target, with a linearity coefficient of 0.99 (Fig. 4c).

**Fig. 5:**
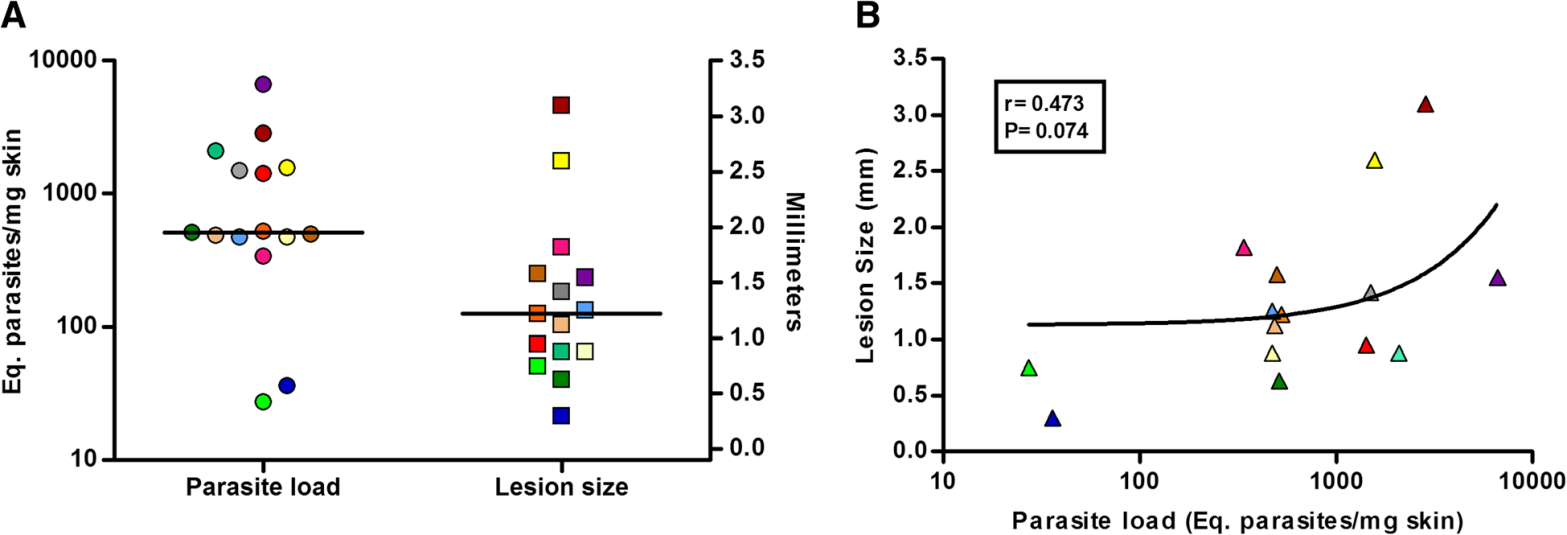
Parasite load quantitation in skin lesion of golden hamsters infected with *Leishmania* (*V*.) *braziliensis.* In **a**, the graphic shows the parasite load (*circles*) and lesion size (*squares*) of golden hamsters infected with 10^5^ *Leishmania* (*V*.) *braziliensis* promastigotes, 110 days post-infection. Parasite load is expressed in parasite equivalents/hamster skin mass (mg) and lesion size is expressed in millimeters. The *horizontal lines* represent the median between the values. In **b**, the graphic shows the Spearman correlation between parasite load and lesion size. The *r* and *P* values are indicated in the graphic. Each animal is represented by a different colour in the graphics (*n* = 15)

In order to validate the qPCR assay, the quantitation of parasite load in skin samples with different lesions sizes were performed in 15 infected golden hamster samples. In 110 days post-infection, animals presented lesions with aspects ranging from small nodules to ulcerated lesions. A range of 27.2 to 6715 parasites/mg skin equivalents (Fig. 5a) was observed, with a median of 553.4 [416.7–1504.0] and mean = 1337. On the other hand, lesion size varied from 0.3 to 3.1 mm, with a mean of 1.34 (± 0.74). Despite the tendency of bigger lesions to present a higher parasite load, the correlation observed was not statistically significant (Fig. 5b).

Taken together, our results represent one important advance in the use of golden hamster as a model for CL study. The methodology of RT-qPCR and qPCR assays using the SYBR Green fluorophore presented high sensitivity and specificity to quantify with precision the cytokines and enzymes related to the immune response, and to estimate parasite load from skin lesions.

## Discussion

The most susceptible mouse strain to *L. braziliensis* infection is BALB/c mice, but skin lesions tend to spontaneously cure within a few weeks [6, 7]. Although hamsters are the most susceptible animals to infection by *L. braziliensis*, studies conducted in this model are hampered by the lack availability of reagents [20]. The usual tools to evaluate the immune response in experimental models are not useful in the hamster model as evidenced by Zivcec and colleagues [25], which tested the cross reactivity of mouse and rat antibodies with the hamster model by using commercially available reagents for ELISA and Luminex. Despite the extensive list of rodent markers analysed, immune compatibility with hamsters was not observed, emphasizing the importance of developing molecular tools, such as RT-qPCR, to evaluate parameters relating to immune responses in this model.

The first data related with the immune response in hamsters experimentally infected with *Leishmania* was in the model of visceral leishmaniasis, obtained by molecular approaches. The cloning and sequence analysis of hamster genes related to infection with *L. donovani* in this animal model was described, revealing a non-polarized immune response profile characterized by an increase in expression of IL-2, IL-12, IFN-ɣ, TNF, IL-10 and TGF-β genes [21, 22]. The same group also published a study about infection of hamsters with *L.* (*V*.) *panamensis*, where, at the site of infection, a type 1 immune response profile (IL12p40, IFN-ɣ) and type 2 (IL-10 and TGF-β) in the early and chronic phase of infection was observed [9]. Later, it was also observed, using TaqMan assays, that gene expression of cytokines and chemokines, after seven days of infection with the same *Leishmania* species, presented a mixed pattern of response, characterized by increased expression of IFN-ɣ and IL12p40, as well as IL-4, IL-10, IL- 13, and IL-21 [18]. In the latter, in order to compensate the low basal expression of certain cytokines in skin, authors used baby hamster kidney (BHK) cells as the reaction calibrators. In a previous study from our group, Ribeiro-Romão and colleagues [11] analysed the gene expression of IFN-ɣ and IL-10 in hamsters infected with 10^4^, 10^5^ and 10^6^ promastigotes of *L. braziliensis*. A significant increase in IFN-ɣ expression in skin and lymph node was observed, more expressive in the skin and the up regulation on the IL-10 expression in lymph nodes from animals infected with the higher innoculum. Despite this, no differences were observed in the expression of these cytokines between the different inocula.

Herein, the quantitation of IFN-ɣ, TNF, IL-6, IL-10, TFG-β, IL-4, iNOS and arginase gene expression demonstrated the applicability of a SYBR Green assay for the quantitation of gene expression of a panel of immunological markers in skin and lymph node from hamsters infected with *L. braziliensis*. Furthermore, in uninfected animals, we observed a higher basal expression of targets IFN-ɣ, TGF-β, IL-4 and TNF in the lymph node compared with the skin, as shown in Fig. 2. This can be attributed to the fact that lymph node is a lymphoid organ with a higher concentration of T cells and macrophages, cells associated with cytokine production.

The present study showed the upregulation in the expression of all target genes measured in skin and lymph node from animals after 110 days of infection, in comparison to uninfected animals. Interestingly, the expression of some cytokines and molecules had striking differences between different tissues in infected animals. For example, IFN-ɣ skin expression was twenty times higher than the expression in lymph nodes, as well as TNF expression in skin reached two times the expression observed in lymph nodes. Finally, IL-10 expression in skin was almost 1000 times greater than the expression in lymph node. Besides, we also observed a large variation in expression of some targets in infected animals, which can be attributed to the outbred model, presenting different clinical and immunological manifestations, as occurs in the human disease.

Therefore, our results showed a mixed type 1 and type 2 pattern of immune response in this phase of infection. A similar pattern was observed in the *L. panamensis* infection [9, 18] and in the *L. donovani* hamster model [21, 22], indicating that this balanced type of response can also contribute to the chronicity of the disease observed in hamsters infected with *L. braziliensis*. Of note, the resistance described in the BALB/c murine model infected by *L. braziliensis* is attributed to the polarization to Th1 immune response, with increased production of IFN-ɣ and the absence of IL-4 [6]. A mixed response is also observed in other diseases in the hamster model, such as leptospirosis [30] and yellow fever [31], suggesting that the susceptibility to infection in this model by many microorganisms can be attributed to the immune response profile.

Effector T cells, that are capable of producing distinct cytokines, can migrate to infection sites and drive efficient anti-*Leishmania* immune responses both at the lesion site and in draining lymph nodes. These responses are well driven by Th1 CD4^+^ T cells producing IFN-γ and TNF, in conjunction with regulatory cytokines, like IL-10. Thus, the nature of the CD4^+^ T cell response during active CL and through disease resolution is associated with robust production of inflammatory cytokines, such as TNF and IFN-γ, even in the presence of IL-10, a regulatory cytokine that acts to reduce the production of TNF [32]. In patients with cutaneous leishmaniasis derived from *L. braziliensis* infection, production of IFN-γ and IL-10 was detected in the skin lesions [33]. It is possible that CD4^+^CD25^+^IL-10^+^TGF-β^+^ T cells are involved in the modulation of the effector immune response in the skin lesions induced by *Leishmania* infection in humans. These results strongly suggest that IL-10 and TGF-β are produced by CD4^+^CD25^+^ T cells at the site of *L. braziliensis* infection [34]. In the case of *L. panamensis*, both the human disease and the mouse model present a mixed cytokine production [35]. Alongside IFN-γ, there is a concomitant production of IL-10, IL-13, IL-17, and TNF. This imbalanced/mixed immune response may be partially responsible for disease pathology.

Interestingly, the expression of arginase and iNOS genes in the hamster model of *L. braziliensis* infection observed in the present work is the opposite to that which occurs in the *L. donovani* infection. While in the hamster model of cutaneous leishmaniasis a smaller increase of arginase expression was observed (up to ten times related to non infected controls), in the visceral form a substantial increase (over a thousand times compared with uninfected controls) was demonstrated. The present study showed that in the hamster model of cutaneous leishmaniasis of *L. braziliensis* there was a greater expression of iNOS in skin (around a thousand times compared with uninfected controls), different from that observed in earlier studies, in which expression in spleens of hamsters infected with *L. donovani* was low, similar to uninfected control [22, 26, 36]. Further studies should be carried out to elucidate whether there is an association of this phenomenon with the pathogenesis of the different clinical forms of the disease in the hamster model.

The usual method to quantitate *Leishmania* in infected tissues is the limiting dilution assay (LDA) [37]. However, this technique is laborious; requires time necessary for the growth of the parasite in culture; depends on sterile conditions and may have impaired results when secondary infections occur in injuries; and can be applied only to fresh samples with relatively high parasite loads due to its low sensitivity [38]. On the other hand, qPCR is a method that has a high sensitivity, accuracy and reproducibility for parasite quantitation [39]. In research on leishmaniasis, reports on the use of qPCR have focused mostly on visceral leishmaniasis [40–45], while reports on CL due to *Leishmania* (*Viannia*) species are scanty.

We demonstrated the applicability of an assay to quantify the parasite load even in hamsters with small fragments of infected tissue. The high sensitivity to the quantitation targeting kDNA from *Leishmania* (*Viannia*) subgenus in lesions has also been reported in human trials [46, 47]. So far, this is the first report describing a quantitative real time PCR assay to estimate the parasite load in skin lesions from hamsters with cutaneous leishmaniasis due to *L. braziliensis* infection. In this model, it has been described that animals with larger lesions present an increase in IFN-ɣ gene expression [17]. Despite the great variability of parasite number between animals and that there was a tendency for small parasite loads in minor lesions, the correlation observed was not statistically significant, which may suggest that the lesion size is not influenced only by the parasitism, but also by the tissue damage caused by an exacerbated immune response.

## Conclusions

In this work, we demonstrate the profile of the immune response induced by *L. braziliensis* infection and the parasite load in the hamster model of cutaneous leishmaniasis by using quantitative PCR. Accordingly, this study allows the improvement of immunological approaches in the hamster model, expanding the possibilities for the design of new drugs and vaccine candidates against CL.

## Abbreviations

cDNA, complement DNA; CEUA, Ethics Committee on Animal Use; CL, Cutaneous leishmaniasis; Ct, Threshold cycle; DALYs, disability-adjusted life years; DNA, Deoxyribonucleic acid; FIOCRUZ, Oswaldo Cruz Foundation; GAPDH, Glyceraldehyde-3-Phosphate Dehydrogenase gene; IFN-γ, interferon gamma; IL-10, Interleukin 10; IL-4, Interleukin 4; IL-6, Interleukin 6; iNOS, inducible Nitric Oxide Synthase; kDNA, kinetoplastid DNA; L., Leishmania; LDA, limiting dilution assay; MIQE, Minimum Information for Publication of Quantitative Real-Time PCR Experiments; PBS, phosphate-buffered saline; PCR, Polymerase chain reaction; qPCR, quantitative real time PCR; RNA, Ribonucleic acid; RT-qPCR, quantitative real time PCR with reverse transcription; TGF-β, Tissue growth factor beta; TNF, Tumor necrosis factor

## Additional files

Additional file 1: Table S1. Determination of the primer concentration to the standardization of RT-qPCR assays for gene expression analysis of immunological markers. To obtain the Ct values, the threshold was set at 0.02 in all assays. (DOC 84 kb) Additional file 2: Table S2. Magnitude of amplification of the basal gene expression of immunological markers in skin and lymph node of uninfected golden Hamsters. To obtain the Ct values, the threshold was set at 0.02 in all assays. (DOC 39 kb) Additional file 3: Table S3. Annealing/extension temperature determination to the standardization of qPCR assay for the parasite load quantification. To obtain the Ct values, the threshold was set at 0.02 in all assays. (DOC 31 kb) Additional file 4: Table S4. Determination of the primer concentration to the standardization of qPCR assay for the parasite load quantification. To obtain the Ct values, the threshold was set at 0.02 in all assays. (DOC 33 kb)
