# Supplementary material for: Development of real-time PCR assays for evaluation of immune response and parasite load in golden hamster (Mesocricetus auratus) infected by Leishmania (Viannia) braziliensis
Source: Parasit Vectors. 2016 Jun 27;9:361. doi: 10.1186/s13071-016-1647-6 (PMC4924296; doi:10.1186/s13071-016-1647-6)
Supplement: Additional file 1: Table S1. — Determination of the primer concentration to the standardization of RT-qPCR assays for gene expression analysis of immunological markers. To obtain the Ct values, the threshold was set at 0.02 in all assays. (DOC 84 kb) [file 13071_2016_1647_MOESM1_ESM.doc]

Additional file 1: Table S1. Determination of the primer concentration to the standardization of RT-qPCR assays for gene expression analysis of immunological markers. To obtain the Ct values, the threshold was set at 0.02 in all assays.

| Target gene | [Primer] Fw/Rv (nM) | Ct mean | SD | Primer-dimer |
| --- | --- | --- | --- | --- |
| IFN-γ | 50/50 | 32.788 | 0.923 | No |
|  | 100/100 | 31.807 | 0.391 | No |
|  | 200/200 | 32.236 | 0.694 | No |
|  | 300/300 | 33.149 | 0.631 | Yes |
| TNF | 50/50 | 25.408 | 1.491 | No |
|  | 100/100 | 23.642 | 0.013 | No |
|  | 200/200 | 23.461 | 0.053 | No |
|  | 300/300 | 23.858 | 0.090 | No |
| IL-6 | 50/50 | 28.175 | 0.569 | No |
|  | 100/100 | 28.289 | 0.460 | No |
|  | 200/200 | 28.614 | 0.018 | No |
|  | 300/300 | 28.706 | 0.202 | No |
| iNOS | 50/50 | 33.996 | 0.431 | No |
|  | 100/100 | 32.940 | 0.356 | No |
|  | 200/200 | 34.203 | 0.937 | Yes |
|  | 300/300 | 32.756 | 0.927 | Yes |
| IL-10 | 50/50 | 28.099 | 0.854 | No |
|  | 100/100 | 28.013 | 0.138 | No |
|  | 200/200 | 27.947 | 0.316 | No |
|  | 300/300 | 28.297 | 0.419 | Yes |
| TGF-β | 50/50 | 18.895 | 0.014 | No |
|  | 100/100 | 18.721 | 0.270 | No |
|  | 200/200 | 18.662 | 0.107 | No |
|  | 300/300 | 19.366 | 0.485 | No |
| IL-4 | 50/50 | 35.008 | 0.113 | No |
|  | 100/100 | 32.967 | 1.154 | No |
|  | 200/200 | 31.786 | 1.362 | No |
|  | 300/300 | 32.704 | 0.473 | No |
| Arginase | 50/50 | 19.031 | 0.405 | No |
|  | 100/100 | 18.884 | 0.142 | No |
|  | 200/200 | 18.818 | 0.123 | No |
|  | 300/300 | 18.806 | 0.165 | No |
| GAPDH | 50/50 | 17.547 | 0.243 | No |
|  | 100/100 | 16.764 | 0.051 | No |
|  | 200/200 | 16.343 | 0.078 | No |
|  | 300/300 | 16.622 | 0.142 | No |
| γ- Actin | 50/50 | 15.352 | 0.128 | No |
|  | 100/100 | 15.117 | 0.139 | No |
|  | 200/200 | 15.587 | 0.558 | No |
|  | 300/300 | 14.624 | 0.505 | No |

Ct: Threshold cycle; SD: Standard deviation
