# Supplementary material for: Development of real-time PCR assays for evaluation of immune response and parasite load in golden hamster (Mesocricetus auratus) infected by Leishmania (Viannia) braziliensis
Source: Parasit Vectors. 2016 Jun 27;9:361. doi: 10.1186/s13071-016-1647-6 (PMC4924296; doi:10.1186/s13071-016-1647-6)
Supplement: Additional file 2: Table S2. — Magnitude of amplification of the basal gene expression of immunological markers in skin and lymph node of uninfected golden Hamsters. To obtain the Ct values, the threshold was set at 0.02 in all assays. (DOC 39 kb) [file 13071_2016_1647_MOESM2_ESM.doc]

Additional file 2: Table S2 Magnitude of amplification of the basal gene expression of immunological markers in skin and lymph node of uninfected golden Hamsters. To obtain the Ct values, the threshold was set at 0.02 in all assays.

| Skin | | | Lymph node | |
| --- | --- | --- | --- | --- |
| Target gene | CT mean | SD | CT mean | SD |
| IFN-γ | 36.276 | 0.295 | 24.771 | 0.148 |
| TNF | 23.028 | 0.163 | 20.352 | 0.058 |
| IL-6 | 27.545 | 0.286 | 25.199 | 0.433 |
| iNOS | 31.942 | 1.772 | 32.824 | 0.784 |
| IL-10 | 35.005 | 0.977 | 24.356 | 0.398 |
| TGF-β | 19.426 | 0.102 | 16.507 | 0.367 |
| IL-4 | 29.762 | 0.345 | 24.817 | 0.023 |
| Arginase | 18.613 | 0.060 | 19.633 | 0.110 |

Ct: Threshold cycle; SD: Standard deviation
