# Supplementary material for: Development of real-time PCR assays for evaluation of immune response and parasite load in golden hamster (Mesocricetus auratus) infected by Leishmania (Viannia) braziliensis
Source: Parasit Vectors. 2016 Jun 27;9:361. doi: 10.1186/s13071-016-1647-6 (PMC4924296; doi:10.1186/s13071-016-1647-6)
Supplement: Additional file 4: Table S4. — Determination of the primer concentration to the standardization of qPCR assay for the parasite load quantification. To obtain the Ct values, the threshold was set at 0.02 in all assays. (DOC 33 kb) [file 13071_2016_1647_MOESM4_ESM.doc]

Additional file 4: Table S4. Determination of the primer concentration to the standardization of qPCR assay for the parasite load quantification. To obtain the Ct values, the threshold was set at 0.02 in all assays.

| [Primer] Fw/Rv (nM) | Ct mean | SD | Primer-dimer |
| --- | --- | --- | --- |
| 300/300 | 20.935 | 0.657 | No |
| 200/200 | 20.401 | 0.096 | No |
| 100/100 | 20.338 | 0.282 | No |
| 50/50 | 19.631 | 0.080 | No |
| 25/25 | 19.994 | 0.076 | No |
| 10/10 | 20.046 | 0.070 | No |

Ct: Threshold cycle; SD: Standard deviation
